# Dual-BERT adversarial learning for Hausa user-generated text normalization

**DOI:** 10.3389/frai.2026.1905680

**Published:** 2026-09-18

**Authors:** Sulaimon Adebayo Bashir, Abubakar Ahmad Aliero, Amina Gogo Tafida, Mukhtar Ibrahim Hussaini, Nur Syadhila Che Lah, Maged Nasser, Yunusa Adamu Bena

**Affiliations:** 1 Department of Computer Science, School of Information & Communication Technology, Federal University of Technology, Minna, Nigeria; 2 Department of Computer Science, Faculty of Computing, Abdullahi Fodio University of Science and Technology, Aliero, Nigeria; 3 Department of General Studies, Federal University of Technology, Minna, Nigeria; 4 Department of Computer Science, Rayhaan University, Birnin Kebbi, Nigeria; 5 Department of Computing, Universiti Teknologi PETRONAS, Seri Iskandar, Malaysia; 6 Faculty of Engineering, Kebbi State University of Science and Technology, Aliero, Nigeria

**Keywords:** BERT, GaN, Hausa language, neural network, text normalization, under-resourced languages, user-generated content

## Abstract

Text normalization (TN) is a crucial preprocessing task in Natural Language Processing (NLP) that enables the conversion of noisy, informal texts into standardized forms suitable for downstream applications, such as machine translation, speech recognition, text-to-speech systems, sentiment analysis, and information retrieval. User-Generated Content (UGC) from social media and messaging platforms often contains irregularities such as abbreviations, phonetic spellings, code-switching, and orthographic inconsistencies. These irregularities have significant implications, especially for low-resourced languages like Hausa, which have limited annotated corpora and computational resources. Existing rule-based and statistical methods struggle in their performance with Hausa text because of spelling variations, code-mixing, abbreviations, and contextual ambiguities. In contrast, neural networks like transformers require a large dataset for efficient training; however, the lack of specialized resources like a parallel corpus further worsens the inefficiency of current models in handling Hausa UGC. To address these gaps, two corpora were developed: a Hausa UGC corpus collected from Twitter and WhatsApp, and a standard Hausa corpus consisting of 27,847 sentences each. The UGC corpus was analyzed to identify Out-of-Vocabulary (OOV) words, their features, and usage patterns. A Dual-BERT Adversarial Network model was then designed and trained to map non-standard Hausa words into their canonical forms accurately. The developed model integrates dual-BERT within an adversarial learning framework, enabling it to capture both the semantic and contextual relationships between informal and standard text. One BERT processes noisy, user-generated Hausa input, while the other learns from standard Hausa sentences, allowing the system to model bidirectional correspondences between the two linguistic forms. Their representations are fused and refined through a generator-discriminator architecture, where the generator produces normalized outputs and the discriminator enforces contextual correctness by distinguishing genuine standard text from generated ones. This adversarial dual-BERT design enhances robustness, contextual awareness, and generalization, making the model highly effective for transforming inconsistent Hausa UGC into standardized text suitable for downstream NLP applications. Experimental results demonstrate that the proposed model achieved an Exact Match Accuracy of 83.99%, BLEU score of 0.7534, WER of 12.02%, and CER of 7.03%, outperforming the evaluated baseline models across all reported metrics. Qualitative analysis further shows that the model effectively normalizes abbreviations, phonetic spellings, character elongations, and many Hausa-English code-mixed expressions, while insertion and deletion errors remain comparatively more challenging. The primary contributions of this work are (i) the construction of a manually annotated parallel corpus for Hausa user-generated text normalization, (ii) the development of a Dual-BERT adversarial framework for context-aware normalization in a low-resource setting, and (iii) a comprehensive evaluation demonstrating the effectiveness of the proposed approach for improving the quality of Hausa user-generated text and supporting downstream NLP applications.

## 1 Introduction

Text normalization (TN) is the process of transforming non-standard or informal text into its canonical or standard form. It is a fundamental preprocessing task for numerous Natural Language Processing (NLP) applications, including machine translation, sentiment analysis, information extraction (IE), information retrieval (IR), automatic speech recognition (ASR), and text-to-speech (TTS) systems (Islam et al., 2024; Sproat and Jaitly, 2016). These downstream applications rely on clean and standardized textual input; however, user-generated content (UGC) collected from social media and online communication platforms is typically noisy and contains abbreviations, acronyms, phonetic spellings, typographical errors, character elongations, and code-mixed expressions (Maghfur et al., 2021; Mehmood et al., 2020; Muhammad et al., 2025).

The rapid growth of mobile communication and social media platforms such as Twitter, Facebook, and WhatsApp has significantly increased the volume of informal language, particularly for low-resource African languages such as Hausa (Abdullahi et al., 2024; Zandam et al., 2023). Users frequently deviate from standard orthographic conventions to communicate more quickly, overcome character limitations, or express emotion. Consequently, noisy UGC has become a major obstacle to developing reliable NLP applications because these irregular linguistic forms reduce the performance of downstream systems. Text normalization aims to resolve these inconsistencies by converting noisy expressions into standardized forms while preserving their semantic meaning. This process is particularly important for low-resource languages such as Hausa, whose unique morphological and phonological characteristics make it difficult to directly transfer normalization models developed for high-resource languages (Rahate and Chandak, 2019; Veliz et al., 2021; Jimoh et al., 2025).

Hausa user-generated content presents numerous normalization challenges, including spelling errors, abbreviations, acronyms, phonetic substitutions, character elongations, insertion and deletion errors, typographical errors, and Hausa–English code-mixed expressions (Sproat and Jaitly, 2016; Abdullahi et al., 2024; Maitama et al., 2014; Zupon et al., 2021; Muhammad et al., 2022). These forms of linguistic variation generate large numbers of out-of-vocabulary (OOV) words that conventional NLP systems cannot effectively process, leading to reduced performance in applications such as machine translation, named entity recognition, sentiment analysis, information retrieval, and text-to-speech synthesis (Dai et al., 2022). A major challenge in Hausa text normalization is that identical non-standard expressions may require different normalized forms depending on their contextual usage. For example, abbreviations, phonetic spellings, and code-mixed expressions can only be interpreted correctly by considering both semantic and syntactic context. Therefore, effective normalization requires models capable of learning contextual representations rather than relying solely on lexical matching or handcrafted linguistic rules (Sproat and Jaitly, 2016; Han et al., 2013).

Existing Hausa text normalization research remains limited. Early studies primarily employed dictionary-based and rule-based approaches to normalize social media text and correct spelling errors (Maitama et al., 2014; Salifou and Harouna, 2014). Although these methods perform adequately for predefined vocabularies, they cannot effectively generalize to unseen abbreviations, evolving social media vocabulary, contextual ambiguities, or code-mixed expressions (Wali and Nisioi, 2025). Recent advances in Transformer-based language models, particularly multilingual BERT, have demonstrated strong contextual representation learning for numerous NLP tasks. Through transfer learning, multilingual BERT can leverage knowledge acquired from high-resource languages and adapt it to low-resource languages such as Hausa, thereby improving contextual understanding despite limited annotated data (Jimoh et al., 2025). However, existing Hausa normalization studies have not fully exploited contextual Transformer representations combined with adversarial learning to improve the robustness and linguistic consistency of normalized user-generated content.

While the global challenge of text normalization is well-established, the specific case of Hausa presents a unique set of challenges, including unique orthographic variations, code-mixing, and a lack of large-scale resources that are only beginning to be addressed with modern NLP techniques. To address these challenges, we propose a Dual-BERT Adversarial Network that leverages two multilingual BERT encoders to learn contextual representations from both noisy and normalized Hausa text. The bidirectional self-attention mechanism enables the model to capture semantic and syntactic dependencies across entire sentences, allowing ambiguous abbreviations, phonetic spellings, and code-mixed expressions to be interpreted according to their surrounding context. Adversarial learning further improves robustness by encouraging the generator to produce normalized outputs that closely resemble authentic standard Hausa text.

The remainder of this paper is organized as follows. Section 2 reviews existing studies on text normalization, with particular emphasis on rule-based, statistical, neural, and Transformer-based approaches for low-resource languages. Section 3 describes the corpus development, annotation methodology, and ethical considerations. Section 4 presents the proposed Dual-BERT Adversarial Network and its training procedure. Section 5 details the experimental setup, evaluation methodology, quantitative results, and qualitative error analysis. Finally, Section 6 concludes the paper by summarizing the main findings, discussing the study’s limitations, and outlining directions for future research.

## 2 Related works

Text normalization is the process of converting raw, unstructured text into a standardized or canonical form. This transformation is a fundamental component in natural language processing (NLP) and text analysis, as it ensures textual consistency and reduces linguistic variability, thereby enabling more accurate and efficient computational processing. In real-world texts, particularly those derived from user-generated content (UGC), various elements such as numerals, abbreviations, acronyms, dates, currency expressions, and other unconventional formats appear frequently. These out-of-vocabulary (OOV) items are typically not captured in standard dictionaries, posing challenges to NLP systems. As a crucial preprocessing step, text normalization addresses these challenges by converting noisy, informal inputs into a uniform structure that NLP models can process more effectively.

The primary objective of text normalization is to map different textual representations of the same concept to a consistent format, making downstream tasks such as sentiment analysis, machine translation, text classification, and information retrieval more reliable. The specific techniques used for normalization vary depending on the NLP task, data domain, and the architecture of the model being employed. This section provides an overview of the key theories and prior research efforts related to text normalization, including challenges in processing noisy text, techniques for error detection and correction, and strategies for transforming and standardizing textual data.

Initial research in the field primarily adopted rule-based approaches. These methods rely on predefined sets of linguistic rules and manually curated mappings to convert non-standard words into their standard equivalents. Although rule-based systems often produce interpretable and accurate results, they require significant manual effort, are time-consuming, and do not scale well with diverse and dynamic text input. For example, Ariffin and Tiun (Ariffin and Tiun, 2020) proposed a rule-based normalization model tailored for Malay social media text, which maps non-standard entries to their correct forms. Similarly, Ahmad et al. (2021) introduced a five-stage modular system for normalizing SMS texts, utilizing a word-error-rate approach with operations such as insertions, deletions, and substitutions. Their system compared incoming text against a structured database and performed normalization accordingly.

Beyond rule-based systems, statistical methods have played a pivotal role in both text normalization and machine translation. Ansari et al. (2017) introduced a statistical model using an optimization-based nearest neighbor matching technique, treating normalization as a similarity matching problem. Their approach combined string-level, phonetic, and contextual similarity measures to align OOV terms with in-vocabulary (IV) counterparts. Evaluations using benchmark datasets demonstrated that parameter tuning on relatively small labeled datasets could yield state-of-the-art performance.

In a related study focused on Thai language normalization, Poolsukkho and Kongkachandra (2018) developed an algorithm targeting Twitter messages using an International Phonetic Alphabet (IPA)-based similarity metric. Their pipeline consisted of multiple components, including OOV detection, phonetic transcription, similarity computation via Levenshtein distance, and candidate word matching. The algorithm achieved a notable accuracy of 79.03%, outperforming the traditional dictionary-based method, LextoPlus, which recorded an accuracy of only 24.19%.

While statistical approaches have shown considerable promise, recent advancements in neural network models have significantly reshaped the landscape of text normalization research. These models enable the automatic learning of complex patterns from data, eliminating the need for manual rule creation or handcrafted features. Lourentzou et al. (2019) proposed a hybrid word-character attention-based encoder-decoder model for social media text normalization. Their end-to-end sequence-to-sequence (Seq2Seq) model effectively handled informal, noisy text, which traditional models trained on formal corpora often fail to process correctly.

Further innovations include multilevel text normalization models that integrate character, word, and sentence-level processing using neural encoders and decoders. Ruzsics and Samardžić (2019) demonstrated how incorporating part-of-speech information, dictionary lookups, and multi-source features into a unified Seq2Seq framework enhanced performance across tasks like lemmatization, canonical segmentation, and orthographic normalization.

Zhang et al. (2019) extended neural approaches to speech-related applications, proposing a series of neural models based on bidirectional recurrent neural networks (BRNNs) with attention mechanisms. These models excelled in converting text for speech applications, handling a variety of normalization tasks while mitigating issues such as catastrophic errors. In a similar direction, Huang et al. (2020) introduced the Local Attention Text Normalization (LATN) model, a deep learning architecture that combines recurrent neural networks with local attention. Their results showed improved accuracy and reduced computational overhead compared to existing approaches, highlighting the value of task-specific deep learning models.

The growing influence of neural networks in text normalization is further evidenced by works such as Kawamura et al. (2020), who developed a model that considers both character-level and phonetic similarity. Their results, measured using F1 scores, demonstrated improved normalization performance compared to baseline models. Additionally, various architectures such as Deep Neural Networks (DNN), Recurrent Neural Networks (RNN) (Makarov and Clematide, 2020), and Long Short-Term Memory networks (LSTM) (Higashiyama et al., 2021) have been explored for their efficacy in learning robust normalization mappings across different languages and text domains. The theoretical foundations of text normalization have evolved from rule-based and statistical methods to sophisticated neural network architectures. These advancements have enabled more accurate and scalable solutions, particularly for handling noisy, informal, and multilingual content in modern communication platforms. As the volume and diversity of user-generated content continue to grow, especially in low-resource languages like Hausa, there remains a strong need for further exploration and adaptation of these theoretical models to new linguistic contexts. Table 1 provide the summary and comparison of the existing normalization approaches discussed Section 2.

**Table 1:** Comparison of existing text normalization approaches.

Authors	Language	Method	Dataset	Evaluation metrics	Strengths	Limitations
Ariffin and Tiun (2020)	Malay	Rule-based	Social media	Accuracy	Interpretable	Poor scalability
Ahmad et al. (2021)	SMS	Rule-based	SMS corpus	WER	Effective for predefined rules	Cannot handle unseen patterns
Ansari et al. (2017)	English	Statistical nearest neighbour	Benchmark dataset	Accuracy	Handles lexical similarity	Requires handcrafted features
Poolsukkho and Kongkachandra (2018)	Thai	IPA + Levenshtein	Twitter	Accuracy	Good phonetic matching	Weak contextual modeling
Lourentzou et al. (2019)	English	Seq2Seq + Attention	Social media	BLEU	End-to-end learning	Requires large datasets
Zhang et al. (2019)	English	BRNN + Attention	Speech normalization	Accuracy	Good sequence modeling	Computationally expensive
Huang et al. (2020)	Chinese	Local Attention	Text normalization	Accuracy	Efficient local context	Limited multilingual applicability
Maitama et al. (2014)	Hausa	Dictionary-based	Facebook	Accuracy	First Hausa normalization study	Limited vocabulary coverage
Salifou and Harouna (2014)	Hausa	Dictionary/Spell checker	Hausa corpus	Accuracy	Corrects spelling errors	Cannot resolve contextual ambiguity
Proposed	Hausa	Dual-BERT + Adversarial Learning	Hausa UGC	EM, BLEU, WER, CER	Context-aware, robust, adversarial optimization	Discussed in Section 5

Although existing studies have made significant progress in text normalization, several limitations remain. Rule-based systems rely heavily on handcrafted linguistic resources and cannot generalize to unseen abbreviations or spelling variations. Statistical methods improve flexibility but depend on manually engineered similarity measures and remain sensitive to noisy user-generated content. Recent Neural Network approaches, including sequence-to-sequence and Transformer models, provide stronger contextual representations but generally require large annotated corpora and have been developed primarily for high-resource languages. Existing Hausa normalization studies are largely dictionary-based and therefore struggle with code-mixing, contextual ambiguity, phonetic spellings, and evolving social media vocabulary. Furthermore, none of the reviewed Hausa studies combine contextual transformer representations with adversarial learning to improve normalization robustness. To address these limitations, this study proposes a Dual-BERT Adversarial Network that combines multilingual contextual representations with adversarial optimization. The dual-BERT architecture enables contextual encoding of both noisy and normalized Hausa text, while adversarial learning improves robustness and linguistic consistency. Unlike previous Hausa normalization approaches, the proposed framework is designed to effectively normalize abbreviations, phonetic spellings, character elongations, and code-mixed expressions within low-resource user-generated content.

## 3 Dataset

The Hausa User-Generated Content (UGC) corpus comprises informal textual data sourced from popular social media platforms where Hausa is actively used. This includes publicly available posts, such as tweets and WhatsApp messages, which are rich in non-standard linguistic forms, including slang, abbreviations, acronyms, and various informal expressions typical of digital communication environments. To ensure linguistic representativeness, the compilation of this corpus followed a structured sampling methodology grounded in four essential criteria, which were prioritized over complete corpus size. This criteria include; Identification of the Target population, Construction of a Sampling Frame, Selection of Sampling Technique, and Data Collection.

The corpus is presented in CSV version. This contains 22,822 tweets and 4,759 WhatsApp chats, which are further broken into 27,847 sentences using full stops and line breaks. The tweet data used was public published by Sani et al. (2022) available at: “https://github.com/hausanlp/naijasenti” consist of three (3) different types of tweets, that is, (1) a short text message consisting of only 140 characters sent by a user, which refers to a simple tweet, (2) a retweet, that is, responding to a tweet by another user to the first user (initiator), and (3) a reply, which is commenting or responding to another tweet, the dataset was mainly used for sentiment analysis which consist of unwanted noisy data like URL, Emojis etc. and this dataset was further preprocess before annotation, all personally identifiable information including usernames, telephone numbers, profile names, URLs, emojis, and other sensitive metadata was removed. Only anonymized textual content was retained for research purposes. While the WhatsApp data was manually were collected only from groups whose participants granted permission for research use, some of which were channels personally created for the purpose of data collection such as “Hausa_chat_style_corpus.” The WhatsApp dataset was collected for the period of about 6 month from September, 2024 to March, 2025. From this, a total of 28,369 unique lexicons were identified, representing the breadth of vocabulary within the corpus. Further analysis revealed 3,492 out-of-vocabulary (OOV) words, highlighting the prevalence of non-standard forms and noisy spellings in Hausa UGC. Additionally, 2,993 English lexicons were identified, reflecting the significant influence of code-mixing practices among Hausa speakers in digital communication. The corpus primarily represents standard written Hausa together with naturally occurring dialectal variations found in Northern Nigeria.

Tables 2–4 present representative samples from the datasets used in this study. Table 2 illustrates examples of the collected Hausa Twitter posts, Table 3 presents sample WhatsApp messages, while Table 4 shows the corresponding manually annotated parallel corpus containing the original unnormalized text, its normalized version, and the English translation. These examples demonstrate the diversity of user-generated Hausa text and the annotation process used to construct the parallel corpus for model training and evaluation.

**Table 2:** Sample of tweeter dataset.

S/No	Hausa tweet data	Meaning
1.	@user Allah ya tsare mana gwamnan talakawa🙏🏾positive	May God protect the governor of the masses
2.	@user Allah ya kai rahmarsa kabarin ta 🤲🤲🤲😭😭 Uwa ga gimbiyar👑👑👑 Alhanislam Hauwa Mainapositive	May God forgive the soul Hauwa Maina, the mother of princesses Alhanislam
3.	@user This was happened today @user Dan musa LGA. Allah ya kawomana dauki Amin😭😭positive	This happened today at Danmusa LGA. May God come to our rescue, amin
4.	@user Allah kaki yayemu baki daya Amin🙏🙏positive	May God protect us all, Amin.
5.	@user Munayin la’akari @user kuma Wallahi mun shaida. Maganar Gaskiya kana qoqari amma kuma tattare dakai akwai naqasu. Munayi maka addu’ah ALLAH SWT ya lurar dakai Kurakuranka, sannan kuma yabaka ikon sauke nauyin daya nauyaya awuyanka 🙏positive	We have realised and witnessed the truth of the matter you are trying but still you have some weakness. We pray may God guide you against your shortcomings and give you the wisdom to delivered.

**Table 3:** Sample of WhatsApp dataset.

S/No	Hausa WhatsApp data	Meaning
1.	Jiya da daddre mukje kasuwa	We went to market last night
2.	Naji kayi shiru ko kaima ka mntane	You keep quit or you have forgotten
3.	Mnyi sbuwar mota alhj	Alhaji we bought a new car
4.	Allah ya shiryeku mata	May God guide you ladies
5.	Kanason asan kananan kenan	You want them to know you are around?

**Table 4:** Sample of annotated dataset.

S/No	Unnormalised data	Normalised data	Meaning
1.	jiya da daddre mukje kasuwa	Jiya da daddare mukaje kasuwa	We went to market last night
2.	naji kayi shiru ko kaima ka mntane	Naji kayi shiru ko kaima ka manta ne	You keep quit or you have forgotten
3.	mnyi sbuwar mota alhj	Munyi sabuwar mota Alhaji	Alhaji we bought a new car
4.	haqiqa kayi matukar kokari	hakika kayi matukar kokari	You have really tried
5.	allah miki albarkah	Allah yayi maki albarka	May God bless you
6.	allah ya karama makan lpy	allah ya karama maka lafiya	May God bless you with good health
7.	allah ya tabbatar da gsky	Allah ya tabbatar da gaskiya	May God reveal the truth
8.	Ni zan iya krba gsky.	Nima zan iya karba gaskiya	Truth, I can equally collect
9.	Son xuciya ne kawai	Soon zuciya ne kawai	It is just a selfish interest
10.	Wllhi ta birgeni matuqa	Wallahi ta birgeni matuka	She has really impressed me

## 4 Proposed framework

This research proposed development of a Dual-BERT Adversarial Network model for Hausa UGC text normalization. The model is motivated by the need to address the high variability, noise, and presence of non-standard linguistic forms within Hausa UGC, which limit the performance of traditional rule-based or purely statistical approaches. The Dual-BERT Adversarial Network architecture combines the representational strength of transformer-based language models with the adversarial learning framework of GANs. Two pre-trained BERT encoders are employed in parallel: one to normalize the noisy, non-standard Hausa text, and the other to validate the normalized reference text. Their joint representations are fed into a generator network, which attempts to produce a normalized Hausa text that is both semantically faithful to the input and stylistically consistent with standard Hausa conventions.

To ensure the robustness of normalization, a discriminator network evaluates the generated outputs by distinguishing between real normalized text (from the gold standard corpus) and synthetic normalized text (produced by the generator). Through adversarial training, the generator iteratively improves its ability to produce outputs that the discriminator cannot reliably distinguish from authentic, normalized Hausa texts. The integration of pre-trained multilingual BERT provides deep contextual embeddings that capture Hausa-specific linguistic structures. This enhances the model’s capacity to generalize across various non-standard forms, including abbreviations and phonetic spellings. Furthermore, the GAN framework ensures that the model does not merely memorize maps but learns to generate linguistically plausible and contextually accurate normalizations. The adversarial loss, combined with sequence-level evaluation metrics such as WER, CER, BLEU, and Exact Match, provides a balanced training signal that optimizes both accuracy and fluency of normalized outputs. This innovative design thus positions the Dual-BERT GAN as a powerful approach for low-resource language processing tasks, extending beyond Hausa normalization to broader applications in text cleaning, machine translation pre-processing, and code-mixing resolution. Figure 1 presents the proposed design of the dual-BERT adversarial network framework.

**Figure 1:**
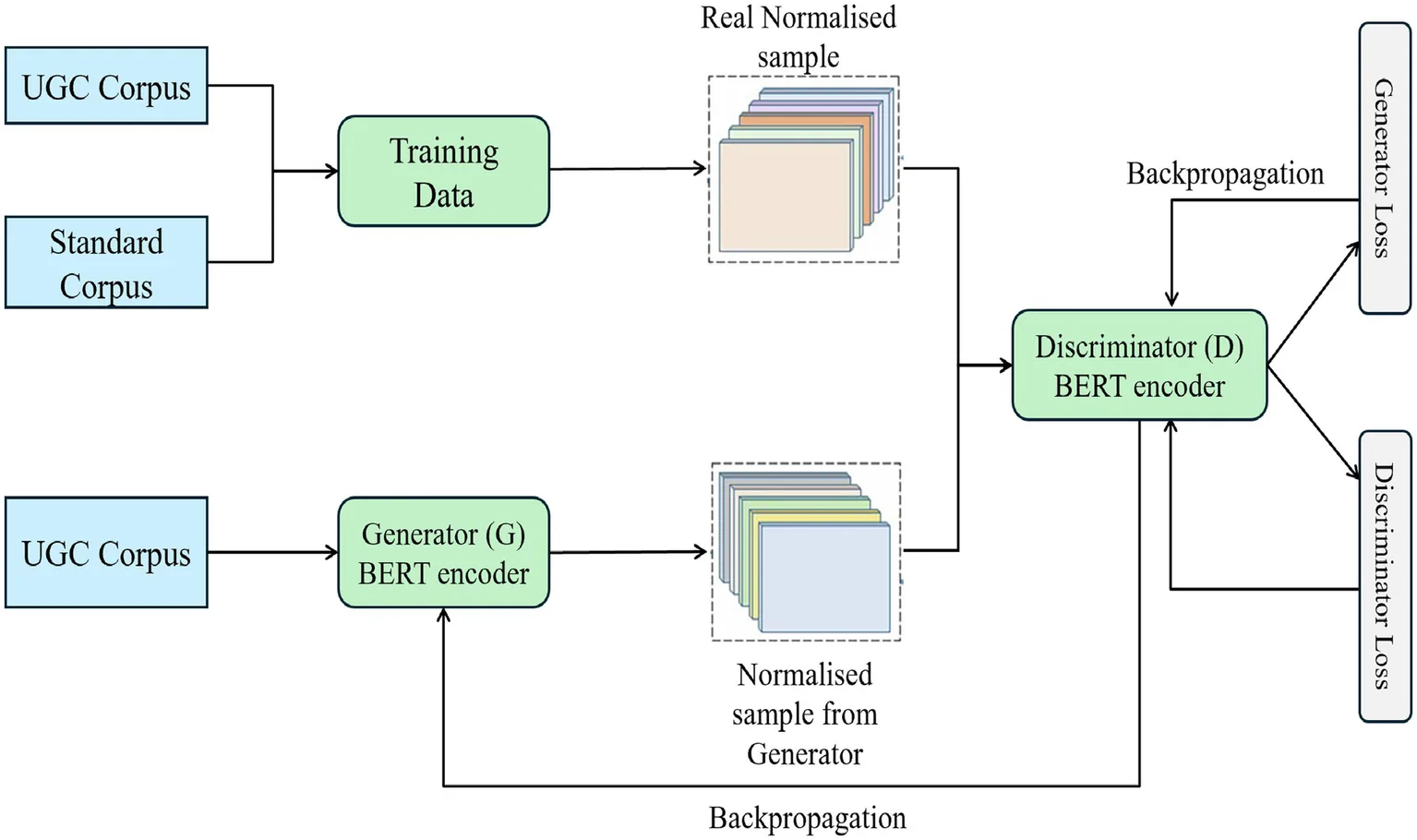
Proposed Hausa text normalization dual-BERT adversarial network model.

The proposed model is designed to convert non-standard (unnormalized) Hausa text into its normalized form using a deep learning approach that integrates BERT with an adversarial training mechanism. The architecture is inspired by the Generative Adversarial Network (GAN) paradigm but applied to natural language processing tasks, particularly context-sensitive text normalization.

At its core, the framework employs a Dual-BERT adversarial architecture comprising two primary components:

- Generator: Responsible for producing normalized text from noisy input. It leverages a pretrained BERT encoder to capture deep contextual information from informal user-generated content effectively.
- Discriminator: Functions as a BERT-based binary classifier that distinguishes between human-generated normalized sentences and those produced by the Generator.

Unlike traditional GANs that utilize simple feed-forward neural networks, this model incorporates pre-trained language models (specifically, BERT) in both the Generator and Discriminator. This enhancement enables the system to better understand and process the syntactic and semantic complexities inherent in Hausa informal and formal texts. The framework is particularly suited for sequence-to-sequence transformation tasks, making it highly effective for handling noisy user-generated Hausa text across platforms such as Twitter and WhatsApp.

### 4.1 Generator network (G)

The Generator is responsible for converting noisy or non-standard Hausa text into its grammatically correct and orthographically accurate counterpart. It is based on a fine-tuning BERT encoder followed by a linear transformation layer that maps BERT’s hidden states to vocabulary logits. The generator’s role is to transform noisy inputs into normalized outputs. It effectively captures the contextual prompt using the pre-trained multilingual BERT. Architecturally, it comprises of a pretrained BERT encoder (BERT-base-multilingual-cased), which captures rich contextual representations of input tokens across multiple layers, and a linear output layer that maps the final hidden states of the encoder to vocabulary-sized logits, which are subsequently decoded into tokens representing normalized text. The proposed normalization model is formulated as a context-aware subword prediction task rather than an autoregressive sequence generation problem. Input sentences are first tokenized using the multilingual BERT WordPiece tokenizer, producing aligned subword sequences. Each subword embedding generated by the BERT encoder is independently classified into its normalized counterpart using a linear projection layer over the shared vocabulary. Consequently, the generator predicts normalized subword tokens in parallel while exploiting contextual information from the entire sentence.

During training, the BERT in generator part receives a noisy input sequence (example, a WhatsApp message with abbreviations and phonetic spellings) and learns to output a cleaned version that adheres to standard Hausa linguistic norms. The use of a pre-trained multilingual BERT enables the generator to leverage deep semantic understanding and cross-lingual features, thereby enhancing performance in low-resource settings.

The Generator is responsible for mapping unnormalized input $x^{u}$ to a normalized output ${\hat{x}}^{n}$ that approximates the true normalized text $x^{n}$ . This process comprises of three stages:

#### 4.1.1 BERT encoder

The BERT encoder is the foundational component of the Generator network in the adversarial BERT-based text normalization model. It processes unnormalized input text and converts it into a contextualized representation that captures the semantic meaning of each token in its full sentence context. The Generator employs a pre-trained multilingual BERT encoder, denoted by $E_{G}$ , which is fine-tuned using the unnormalized Hausa text corpus to learn contextual representations of noisy user-generated content.

$$H^{u}=E_{G}\left(x^{u}\right)=\left[h_{1}^{u},h_{1}^{u},\ldots ,h_{1}^{u}\right]\in {\mathbb{R}}^{T\times d}$$ (1)

Where:

- Hu: The contextualized embedding matrix output by the BERT encoder for the input xu.
-
$H_{i}^{u}$ : The embedding vector for token $x_{i}^{u}$ , computed by attending to all other tokens in the sequence using BERT’s self-attention mechanism.
- T: The sequence length, that is number of tokens in the input sentence.
- d: The hidden size, dimensionality of each token embedding vector output by BERT (commonly *d* = 768 for BERT-base).

#### 4.1.2 Transformation layer

The Transformation Layer (denoted by TG) is the decoding component of the Generator (G). It operates directly on the contextual embeddings produced by the BERT encoder and transforms them into a sequence of probability distributions over the vocabulary. Each distribution represents the likelihood of possible output tokens (normalized words) at each position in the sequence.

Let the output of the BERT encoder be Equation (1).

Then the transformation layer TG projects each $h_{i}^{u}$ into a vector of size equal to the vocabulary size |*V*|, and applies the softmax function to produce a probability distribution over the vocabulary at that time step is given by Equation 2:

$${\hat{y}}_{i}\mathrm{Softmax}\left(Wh_{t}^{u}+b\right)\forall t\in \left[1,T\right]$$ (2)

Where:

-
$\hat{y}\in {\mathbb{R}}^{|V|}$ predicted probability distribution over the vocabulary for the token at time step t.
-
$W\in {\mathbb{R}}^{|V|\times d}$ : A learnable weight matrix that projects each embedding from *d*-dimensions to |*V*|-dimensions.
-
$b\in {\mathbb{R}}^{|V|}$ : A learnable bias vector.
-
e : embedding representation.
- SoftMax(·): A function that converts the output logits into a valid probability distribution:

$$\mathrm{Softmax}\left(z_{i}\right)=\frac{e^{z_{i}}}{\sum_{j=1}^{\left|V\right|}e^{z_{j}}}\mathrm{for each}\;i\in \left[1\mathrm{,||,||}V\mathrm{,||}\right]\;$$ (3)

Thus, for each position in the sequence of Equation 3, the model produces a distribution over all possible output tokens, from which the most likely normalized word is selected (example, via greedy decoding or beam search during inference).

Unlike encoder-decoder architectures such as T5 or BART, the proposed model does not perform left-to-right text generation. Instead, normalization is treated as a contextual token transformation problem in which every input token is simultaneously mapped to its normalized representation. The bidirectional contextual representations produced by BERT allow each prediction to exploit both preceding and succeeding context, which is particularly beneficial for resolving abbreviations, phonetic spellings, and ambiguous user-generated expressions.

#### 4.1.3 Generator output

The Generator Output is the final product of the Generator (G) module in the model. It represents the predicted normalized version of the unnormalized input text. The Generator output is obtained by passing the sequence of contextual embeddings produced by the BERT encoder through the Transformation layer, which maps each embedding to a probability distribution over the vocabulary and selects the most likely tokens.

$$\hat{x}=G\left(x^{u}\right)=T_{G}\left(E_{G}\left(x^{u}\right)\right)$$ (4)

This Equation 4 describes the composition of functions involved in producing the normalized output:

$E_{G}\left(x^{u}\right)$ : Applies the BERT encoder to the unnormalized input, generating contextualized hidden states (embeddings) $H^{u}\in {\mathbb{R}}^{T\times d.}$

$T_{G}\left(H^{u}\right)$ : Applies the transformation layer to each hidden state $H_{t}^{u}$ , producing token-level probability distributions over the vocabulary.

$G\left(x^{u}\right)$ : The full Generator function that returns the normalized output sequence by selecting the most probable token at each time step (example, via: ${\hat{x}}^{n}=({\hat{x}}_{1,}^{n}{\hat{x}}_{2,}^{n}I,{\hat{x}}_{T}^{n}$ ).

Where ${\hat{x}}_{t}^{n}={\mathrm{argmax}}_{w\in V}{\hat{y}}_{t}\left[w\right],\mathrm{with}\;{\hat{y}}_{t}\in {\mathbb{R}}^{|V|}$ being the predicted probability distribution at time step t.

#### 4.1.4 Generator supervised loss

The proposed approach performs aligned subword prediction, the input and output sequences maintain identical token positions after WordPiece tokenization. Variable-length normalization is therefore handled at the tokenizer level rather than by the neural architecture itself. When a lexical item is segmented into multiple WordPiece tokens, the model predicts normalized subword units for each position. During post-processing, consecutive predicted subwords are merged to reconstruct the normalized surface form. Character insertions and deletions commonly observed in Hausa user-generated content (for example repeated characters, omitted vowels, and phonetic substitutions) are normalized implicitly through subword prediction. The WordPiece tokenizer decomposes such irregular words into meaningful subword units, allowing the generator to predict the appropriate normalized subwords. The reconstructed output therefore restores the canonical spelling without requiring explicit edit operations. Word-order modification is outside the scope of the proposed normalization task. The developed corpus contains parallel sentence pairs in which normalization primarily consists of orthographic correction, abbreviation expansion, phonetic normalization, and lexical replacement while preserving sentence structure. Consequently, no explicit sequence reordering module is required.

### 4.2 Discriminator network (D)

The Discriminator functions similarly to a BERT-based binary classifier. It is trained to distinguish between human-generated normalized text and the generator’s outputs. The binary cross-entropy loss encourages it to become better at classification, which in turn forces the generator to produce more human-like text. It operates as follows:

- It takes either a real (human-written) or fake (machine-generated) normalized sentence as input.
- Process it through a BERT encoder.
- Passes the encoded features through a binary classification layer that outputs a probability score indicating the likelihood that the input is authentic.

The discriminator is trained using binary cross-entropy loss, penalizing incorrect classifications. As it becomes better at detecting generated text, it provides informative gradients that guide the generator toward producing more fluent and human-like outputs.

Discriminator is the second major component in the adversarial framework. Its role is to evaluate whether a given normalized sentence is:

- Real - directly taken from the true (human-labeled) normalized dataset.
- Fake - generated by the generator G from unnormalized input.

By training on both real and generated samples, the Discriminator improves its ability to detect subtle differences in quality and linguistic structure, thereby encouraging the generator to produce more accurate, human-like normalized text. The discriminator D consists of two main layers:

- BERT Encoder

Another pre-trained BERT model, denoted ED , fine-tuned on normalized text. It transforms the input sequence (either real or generated normalized text) into a representation as in Equation 5:

$$H=E_{D}\left(x\right)=\left[h_{1,}h_{2,I}h_{T}\right]\in {\mathbb{R}}^{T\times d}$$ (5)

Where:

H: Matrix of contextual embeddings for the input tokens.

Ht : Embedding vector of the *t*-th token.

D: Hidden dimension (example, 768 for BERT-base).

T: Sequence length (number of tokens in the input sentence).

ii Classification Layer

A classifier C applied on the [CLS] embedding is passed through a linear classification layer with sigmoid activation as illustrated in Equation 6:

$$D\left(x\right)=\sigma \left(W_{c}h_{\left[\mathrm{CLS}\right]}+b_{c}\right)\in \left[0,1\right]$$ (6)

Where:

D(x): Output probability indicating whether *x* is a real normalized sentence.

Bc: Bias term.

Wc: classification weight matrix

h[CLS] : hidden state of the [CLS] token

*σ*(·): sigmoid function that returns a score in [0,1]

Algorithm 1 introduces the enhanced GAN framework tailored for this study, where the generator is powered by a BERT architecture with a residual connection to retain input context, and the discriminator is streamlined to use only the [CLS] token from a BERT encoder for efficient and accurate classification. The modified training procedure also incorporates gradient clipping, label smoothing, and alternating updates to stabilize the adversarial training process. Together, these enhancements make the Dual-BERT adversarial network model well-suited for context-sensitive text normalization in low-resource languages, such as Hausa, particularly within noisy, user-generated content domains.

Algorithm 1: Training algorithm for Dual-BERT adversarial network framework.

1 **For** number of training iterations do
2  **For** k steps do
3   Sample minibatch of m unnormalized data {z^(1)^,…, *z*^(*m*)^} from prior Data(*z*)
4   Sample minibatch of m normalized data {*x*^(1)^,…, *x*^(*m*)^} from Data(*x*)
5   Generate normalized predictions {x̂^1^, I, x̂^m^} using the Generator:
6    x̂^(i)^←G(*z*^(*i*)^)
7   Prepare labels with smoothing:
8    real_labels = 0.9 (for x^(i)^)
9    fake_labels = 0.1 (for x̂^(i)^)
10   Extract [CLS] token embeddings from BERT for both x^(i)^ and x̂^(i)^11   Update discriminator parameters θ_d_ by ascending the gradient:
12    Using Equation 9
13   Apply gradient clipping on θ_d_ (clipnorm = 1.0)
14  **End** for
15  Sample minibatch of m unnormalized data {z^(1)^,…, *z*^(*m*)^} from prior Data(*z*)
16  Generate normalized outputs x̂^(i)^ = G(*z*^(*i*)^)
17  Update generator parameters θ_g_ by descending the gradient:
18   Using Equation 8 ← alternate to maximize fooling score
19  Apply residual connection post-BERT within Generator:
20   G_out_ ← Residual(G_BERT_(z), z)
21  Apply gradient clipping on θ_g_ (clipnorm = 1.0)
22 **End for**

### 4.3 Adversarial training strategy

In the adversarial BERT-based text normalization model, the generator *G* and discriminator *D* are trained in a dynamic two-player game. This training approach is inspired by the GAN framework, where the generator attempts to produce high-quality normalized text from unnormalized input and the discriminator attempts to distinguish between real normalized text (from the ground truth dataset) and fake normalized text (produced by the generator). The training process is adversarial in nature, meaning both components improve in opposition: the generator learns to “fool” the discriminator, while the discriminator learns to detect whether a sequence is genuine or generated.

During training, the generator does not perform discrete token selection. Instead, it produces a probability distribution over the vocabulary for every subword position using the Softmax function. These continuous probability distributions are used during adversarial optimization, allowing gradients from the discriminator to propagate back through the transformation layer into the BERT encoder. Discrete token selection using argmax decoding is performed only during inference after model optimization has been completed. The discriminator receives two types of normalized sequences. The first consists of gold-standard normalized sentences from the manually annotated corpus. The second consists of normalized predictions produced by the generator. During adversarial optimization, generated outputs are represented by continuous token probability distributions rather than discrete sampled tokens, ensuring end-to-end differentiability.

Because vocabulary probabilities remain continuous, gradients computed by the discriminator can be propagated through the transformation layer and subsequently through the generator BERT encoder using standard backpropagation. Consequently, no reinforcement learning, policy-gradient optimization, Gumbel-Softmax approximation, or straight-through estimator is required. During inference, the probability distribution at each token position is converted into a normalized token using greedy decoding (argmax). Consecutive WordPiece tokens are then merged to reconstruct the final normalized sentence. The full adversarial can be expressed as in Equation 7

$$\underset{G}{\min}\underset{D}{\;\max\;}E_{x^{n}∼D_{n}}\left[\log D\left(x^{n}\right)\right]+E_{x^{u}∼D_{u}}\left[\log\left(1-D\left(G\left(x^{u}\right)\right)\right)\right]$$ (7)

Where:

*x^n^*: Real normalised sentence from the dataset.

*X^u^*: Unnormalised input sentence.

$G\left(x^{u}\right)={\hat{x}}^{n}$ : Normalised text generated by the generator.

D(*x*): Probability that the input *x* is a real normalised sentence.

*D_n_* : The distribution of real training data (that is, real normalised sentences).

i Generator loss

The Equation 8 represent modified generator loss with label smoothing, typically used to stabilized GAN training in our model.

$$\Delta \theta_{d}\frac{1}{m}\overset{m}{\underset{i=1}{\sum }}\log\left(D\left(G\left(z^{\left(i\right)}\right);0.9\right)\right)$$ (8)

Where:

$\theta_{g}$ : generator parameters being updated.

m : Minibatch size.

$z^{\left(i\right)}$ : $i^{\mathrm{th}}$ unnormalised input (or noise sample).

$G\left(z^{\left(i\right)}\right):$ Generator output (predicted normalised text).

$D\left(G\left(z^{\left(i\right)}\right)\right)$ : Discriminator’s probability that the generated sample is real.

0.9 : Smoothed “real” label, Instead of using label = 1, we use 0.9 to prevent overconfidence.

ii Discriminator loss

The Equation 9 represent modified discriminator loss function with a label-smoothing used in the Modified Dual-BERT Adversarial Network.

$$\Delta \theta_{d}\frac{1}{m}\overset{m}{\underset{i=1}{\sum }}\left[\mathrm{logD}\left(x^{\left(I\right)};0.9\right)+\log\left(1-D\left({\hat{x}}^{\left(I\right)};0.1\right)\right)\right]$$ (9)

Where:

$\theta_{d}$ : Discriminator parameters being updated.

m : Minibatch size.

$z^{\left(i\right)}$ : $i^{th}$ unnormalised input (or noise sample).

$\left(x^{\left(i\right)}\right):\;\;$ Real normalised Hausa sentence sampled from the standard corpus.

${\hat{x}}^{i}:\;\;$ Generated (fake) normalised sentence

D (.): Discriminator output - probability that input text is real.

0.1 : Smooth “fake” label, Instead of using label = 0

Figure 2 illustrates the architecture and data flow of the proposed Dual-BERT Adversarial Network Model designed for Hausa text normalization. The system consists of two primary components: the Generator (G) and the Discriminator (D), which operate within an adversarial learning framework to transform noisy Hausa user-generated content (UGC) into standardized text. As shown in the diagram, the process begins with noisy Hausa UGC input, such as *“zamuje kd gb in shaa allah.”* The text is first tokenized into subword units (*z*) before being passed into the Generator (G), a BERT-based encoder network responsible for generating the normalized text. The generator employs a contextual BERT encoder that captures linguistic dependencies and semantic relationships within the input. A residual connection following the encoder ensures that essential semantic features are retained while allowing for orthographic correction and contextual refinement. The generator outputs a normalized Hausa sentence, representing the predicted canonical form of the input.

**Figure 2:**
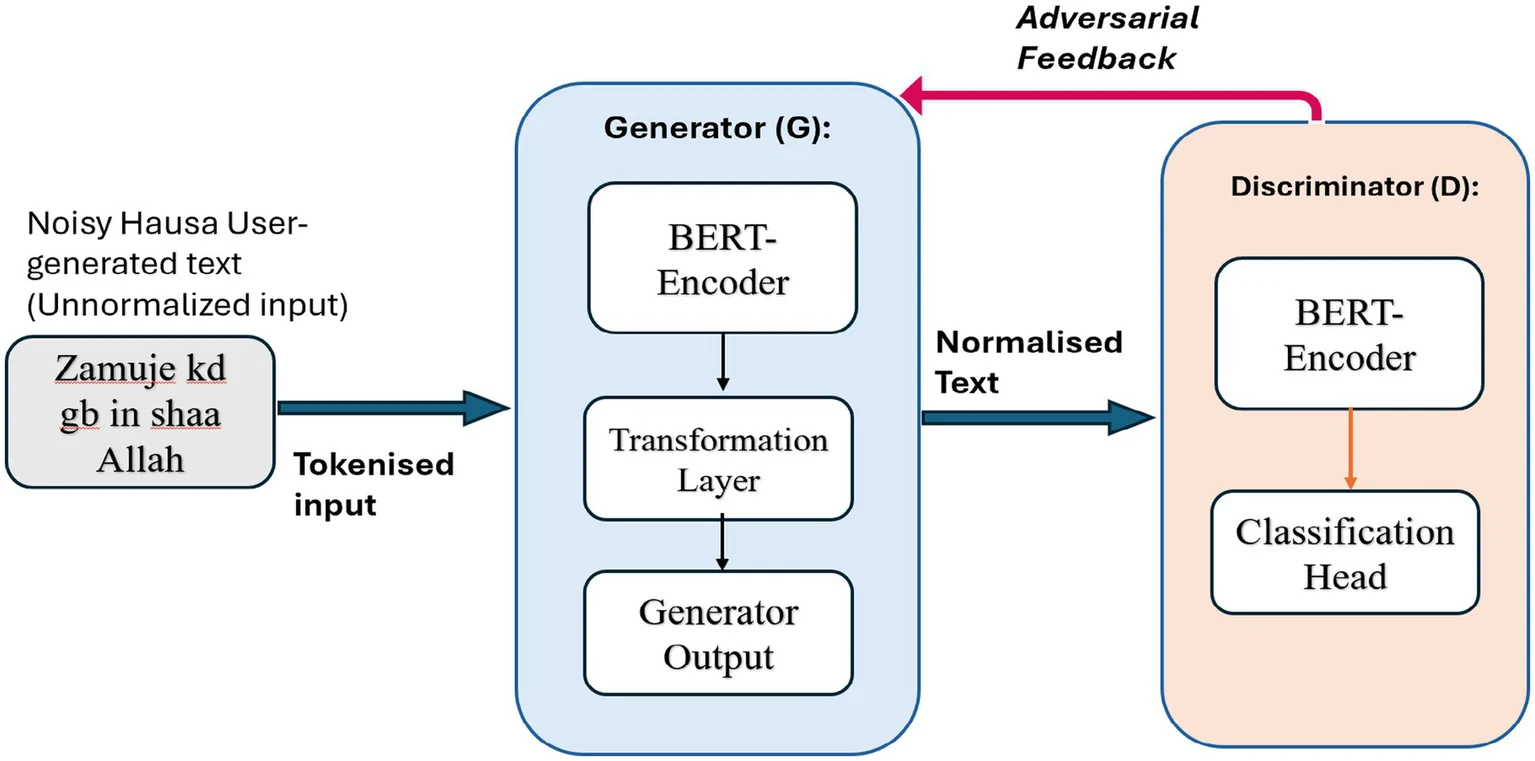
Proposed Hausa text normalization dual-BERT adversarial network model.

The Discriminator (D), also BERT-based, acts as an authenticity classifier that differentiates between real normalized Hausa sentences (from the reference corpus) and outputs generated by the generator. It features a classification head that assigns a probability score indicating whether the text is genuine or machine-produced. Through adversarial training, the discriminator offers feedback and gradient updates to the generator, encouraging it to produce more authentic and contextually accurate normalizations. Additionally, gradient clipping is applied to stabilize training, while the residual connections across both networks preserve semantic meaning and contextual fidelity. This adversarial setup enables the generator to learn high-quality mappings between informal and standard Hausa forms, resulting in improved model robustness, generalization, and linguistic consistency.

### 4.4 Experimental setup

The experimental setup for this research consists of both hardware and software components essential for developing, training, and evaluating the proposed Dual-BERT adversarial network model for Hausa text normalization.

#### 4.4.1 Software environment

All model development and experiments were conducted using Google Collaboratory (Colab), a cloud-based Jupyter notebook environment with GPU support. The Colab runtime was configured with the following key libraries and frameworks:

- Python 3.10 (as the base programming language)
- PyTorch 2.x - for deep learning model implementation
- Transformers (by HuggingFace) – for loading pre-trained BERT models
- TensorFlow 2.x - for compatibility in loss computation and metrics (optional)
- Datasets - for loading and preprocessing custom and benchmark datasets
- NLTK - for BLEU and other linguistic evaluation metrics
- scikit-learn - for computing precision, recall, F1-score, and classification reports
- Matplotlib/Seaborn – for visualizing model loss, performance trends, and metrics
- Tqdm - for tracking training progress via progress bars
- Tokenizers - for managing subword tokenization compatible with BERT
- Pandas and NumPy - for dataset manipulation and statistical summaries

The environment ensured reproducibility and access to GPU acceleration for faster training.

#### 4.4.2 Hardware specifications

Although the training was performed in a cloud environment, the underlying hardware specifications used in Google Colab (Pro version) were approximately:

- GPU: NVIDIA Tesla T4/V100 (allocated based on availability)
- CUDA: 11.x
- CPU: Intel Xeon (virtual machine with 2 cores)
- RAM: Up to 13 GB (RAM)
- Disk Space: Temporary runtime with up to 100 GB (ephemeral)

These resources were sufficient for training the Dual-BERT generator and discriminator over 20 epochs on the Hausa normalization dataset without local deployment.

#### 4.4.3 Data preparation and preprocessing

The dataset used in this study consists of parallel pairs of non-standard (noisy) and standardised Hausa text. These pairs were manually or semi-automatically curated from real-world sources such as Twitter and WhatsApp chat messages, which are rich in informal language constructs, abbreviations, and phonetic spellings. Each noisy entry corresponds directly to a human-normalised version that adheres to the grammatical and orthographic conventions of Standard Hausa.

To prepare the data for supervised learning, the corpus was partitioned into three subsets using a commonly adopted 80–10-10 split ratio:

- Training Set (80%): This subset was used to train the model by enabling it to learn the mapping between noisy and normalised text. The high volume of training data ensures the model generalizes well to diverse input patterns.
- Validation Set (10%): The validation set was used during the training process to monitor the model’s performance and tune hyperparameters. It helped detect overfitting and guided adjustments to learning strategies.
- Test Set (10%): This subset was reserved for final evaluation, providing an unbiased measure of how well the trained model performs on unseen data.

#### 4.4.4 Tokenization process

Before feeding the text data into the neural network model, both noisy and normalised texts underwent a tokenization process using the Bert Tokenizer from the Hugging Face Transformers library. Specifically, the bert-base-multilingual-cased tokenizer was used, which supports the Hausa language among many others. Tokenization is a critical step in natural language processing, particularly when working with BERT-based architecture. It involves splitting raw text into subword units (also called WordPieces) while preserving the semantic integrity of the input. This subword-based approach helps the model manage:

- OOV words, which are common in user-generated Hausa content.
- Morphological complexity, such as inflections or agglutinative word structures.

During tokenization, each sentence was:

- Lowercased to match the tokeniser’s vocabulary structure.
- Truncated or padded to a fixed maximum length (example, 64 tokens) to ensure uniform input dimensions across training batches.
- Encoded into input representations including:
  - input_ids: token indices from the tokenizer’s vocabulary.
  - attention_mask: binary mask indicating padded vs. actual tokens.
  - token_type_ids (optional): used to differentiate sentence pairs in some configurations.

This preprocessing step ensured that all textual data was aligned with the input requirements of the BERT model, facilitating efficient fine-tuning and improved context-sensitive learning.

### 4.5 Model training procedure

The proposed Dual-BERT Adversarial Network was trained using the manually annotated Hausa parallel corpus consisting of noisy user-generated sentences and their corresponding normalized forms. Before training, the corpus was randomly divided into training, validation, and test sets using a 70:15:15 split to ensure unbiased evaluation. All text was preprocessed using the multilingual BERT tokenizer, which segments input into WordPiece subword units to effectively represent out-of-vocabulary words, spelling variations, and noisy user-generated text.

Training was performed by fine-tuning two pretrained multilingual BERT encoders within an adversarial learning framework. During each iteration, a batch of noisy Hausa sentences was first processed by the Generator to produce contextual token representations and normalized token probability distributions through a Softmax layer. During training, these probability distributions were supplied to the Discriminator, allowing gradients to propagate through the Generator during backpropagation. During inference, the final normalized sequence was obtained by selecting the token with the highest probability at each position using greedy decoding.

The Discriminator received both the reference normalized sentences and the Generator outputs and was trained to distinguish between human-normalized and machine-generated text. Simultaneously, the Generator was optimized to minimize a joint objective comprising supervised token classification loss and adversarial loss, whereas the Discriminator was optimized using binary cross-entropy loss. The AdamW optimizer was employed for both networks because of its effectiveness in fine-tuning Transformer-based language models and its ability to provide stable convergence through decoupled weight decay.

To ensure a fair comparison, the baseline Transformer Seq2Seq, multilingual BERT (mBERT), and multilingual Text-to-Text Transfer Transformer (mT5) models were trained using the same Hausa parallel corpus, identical train/validation/test split, identical preprocessing pipeline, comparable optimization settings, and identical evaluation metrics. Each pretrained model employed its native tokenizer (WordPiece for mBERT and SentencePiece for mT5), while maintaining identical learning rate, batch size, number of training epochs, optimizer configuration, and early stopping criteria. Training performance was monitored after every epoch by recording the Generator loss, Discriminator loss, Generator accuracy, and Discriminator accuracy. The model corresponding to the lowest validation loss was retained for final evaluation. The final model was assessed on the held-out test set using Exact Match (EM), BLEU score, Word Error Rate (WER), and Character Error Rate (CER). Table 5 present training hyperparameters used for the all experiment and Table 6 present the mosel size.

**Table 5:** Training hyperparameters used for all experiments.

Hyperparameter	Value
Pretrained model	Multilingual BERT (mBERT Base, cased)
Generator architecture	Multilingual BERT encoder + Linear classification layer
Discriminator architecture	Multilingual BERT encoder + Binary classifier
Optimizer	AdamW
Learning rate	2 × 10^−5^
Weight decay	0.01
Batch size	16
Maximum sequence length	128 tokens
Number of epochs	20
Early stopping	Validation loss
Learning-rate scheduler	Linear decay with warm-up (if used; otherwise omit)
Warm-up ratio	10% (if used; otherwise omit)
Dropout	0.1
Gradient clipping	1.0
Random seed	42
Training/Validation/Test split	70%/15%/15%
Evaluation metrics	Exact Match (EM), BLEU, WER, CER

**Table 6:** Model size.

Model	Approximate number of parameters
Transformer Seq2Seq (6 Encoder + 6 Decoder)	~65–80 million*
mBERT Base	~110 million
mT5 Base	~580 million
Proposed Dual-BERT Adversarial Network	~220 million**

* Depends on vocabulary size and implementation. ** Two multilingual BERT encoders (≈110 M parameters each) plus lightweight Generator and Discriminator classification heads.

## 5 Result and discussion

Training was conducted over 20 full epochs, with both training and validation performance evaluated at the end of each epoch. Table 2 summarizes the progression of key training metrics, including generator and discriminator losses, Exact Match (EM) accuracy, Word Error Rate (WER), Character Error Rate (CER), and BLEU score. During epochs 1 to 5, the model exhibited rapid learning behavior. The generator loss decreased significantly from 9058.82 to 1801.35, reflecting a marked improvement in the model’s ability to produce normalized outputs that mimic the reference standard. Similarly, the Exact Match accuracy increased from 0.1881 to 0.5252, indicating that over 52% of predictions exactly matched the gold-standard normalized forms. The BLEU score, which captures the overlap between predicted and reference sequences, also rose from 0.4794 to 0.7162, further validating the effectiveness of early learning. However, at epoch 6, the model encountered a notable instability, while the generator loss continued to decline to 1577.37, suggesting that the model was still optimizing its outputs performance metrics showed a dramatic collapse. The Exact Match accuracy dropped sharply to 0.0344, WER surged to 1.0391, and CER rose to 0.8972. These anomalies indicate a breakdown in generalization capability, potentially due to overfitting, vanishing gradients, or imbalance in adversarial training dynamics (that is, the generator becoming too aggressive or the discriminator too weak). This period may reflect a phase of mode collapse, where the generator repeatedly outputs similar or degenerate sequences.

Fortunately, the model recovered in epoch 7 to 10, regaining predictive stability. The exact match rose steadily to 0.5950, and BLEU score peaked at 0.7591 by epoch 10. WER and CER also showed corresponding improvements, signifying that the model had overcome the earlier disruption and resumed effective learning. From epoch 11 through epoch 20, the model entered a phase of convergence and stabilization. BLEU scores consistently remained above 0.74, and WER values dropped and stabilized below 0.20. The generator loss showed diminishing changes, hovering around 662–700, while the discriminator loss gradually increased, indicating that the generator output had become increasingly difficult to distinguish from real examples. This stabilization phase confirms that the model had effectively learned to normalize non-standard Hausa inputs, maintaining both robustness and generalization across varying linguistic distortions. The Transformer Seq2Seq baseline consisted of the standard encoder–decoder Transformer architecture comprising six encoder layers and six decoder layers. mBERT consisted of 12 Transformer encoder layers, 12 self-attention heads, a hidden embedding size of 768 dimensions, and approximately 110 million parameters, while mT5 consisted of the multilingual Text-to-Text Transfer Transformer (mT5-Base) with 12 encoder layers, 12 decoder layers, a model dimension of 768, and approximately 580 million parameters. All baseline models were trained on the same Hausa parallel corpus using identical preprocessing, tokenizer, optimizer, batch size, learning rate, early stopping criteria, and evaluation protocol as the proposed model. To ensure a fair comparison, all models used the same train/validation/test split, identical preprocessing pipeline, identical WordPiece vocabulary (where applicable), and identical evaluation metrics.

The performance of the proposed Dual-BERT Adversarial Network Model was evaluated using several intrinsic metrics, including precision, recall, F1-score, Word Error Rate (WER), Character Error Rate (CER), and Bilingual Evaluation Understudy (BLEU). The model achieved 83.99% exact-match accuracy, a BLEU score of 75%, a WER of 12.02%, and a CER of 7.03%, demonstrating strong capability in generating accurate and contextually appropriate standard Hausa text from user-generated input. The F1-score of 84.21% further confirmed the model’s balanced precision-recall performance.

Table 7 summarizes the training performance of the Generator and Discriminator throughout adversarial optimization. The steady improvement in Generator accuracy indicates that the contextual representations learned by the multilingual BERT encoder become increasingly effective at predicting normalized Hausa text. At the same time, the gradual reduction in Generator loss demonstrates improved convergence toward the reference normalized sentences. The Discriminator maintained a balanced classification accuracy throughout training, suggesting that it remained sufficiently challenging to encourage continual improvement of the Generator without dominating the optimization process. This balanced adversarial interaction contributes to stable learning and reduces the risk of mode collapse or premature convergence. In comparative experiments, the Dual-BERT Adversarial Network outperformed rule-based, statistical, and transformer S2S baseline models by an average margin of 9–12% across all metrics. The improvement is attributed to the model’s dual contextual embedding mechanism, which effectively captures both informal and canonical linguistic features. The adversarial feedback loop between the generator and discriminator further enhances the generator’s ability to produce realistic outputs by reducing overfitting and enforcing semantic consistency. The training process showed stable convergence, with both generator and discriminator losses reaching equilibrium after approximately 20 epochs. Analysis of the learning curves indicated consistent improvement in BLEU and F1 scores, validating the robustness of the adversarial optimization.

**Table 7:** Summary of key training metrics progress.

Epoch	Generator loss	Discriminator loss	Exact match	WER	CER	BLEU
1	9058.8228	529.8936	0.1881	0.3981	0.2615	0.4794
2	3673.6671	1073.2284	0.4050	0.2851	0.1646	0.6293
3	2661.5324	1211.6209	0.4394	0.2271	0.1312	0.6745
4	2135.5033	1260.7627	0.4431	0.2919	0.2128	0.6678
5	1801.3493	1281.4857	0.5252	0.2029	0.1285	0.7162
6	1577.3728	1305.5107	0.0344	1.0391	0.8972	0.4194
7	1392.6430	1320.1110	0.4761	0.2184	0.1364	0.7092
8	1262.6583	1332.7836	0.3931	0.2642	0.2019	0.6813
9	1141.1551	1344.0587	0.4174	0.2253	0.1611	0.6981
10	1064.0880	1353.2418	0.6950	0.1592	0.0841	0.7591
11	988.4734	1361.6613	0.5849	0.2495	0.1944	0.6810
12	879.7947	1369.9052	0.7349	0.1846	0.1191	0.7424
13	860.5763	1377.2115	0.6381	0.1904	0.1077	0.7382
14	831.1894	1383.1514	0.5509	0.1967	0.1425	0.7180
15	707.8461	1386.9313	0.7803	0.1680	0.0990	0.7573
16	694.1290	1392.7496	0.6229	0.1211	0.0606	0.7835
17	709.3831	1391.5171	0.8229	0.1566	0.0949	0.7527
18	713.3800	1395.4515	0.5546	0.1329	0.0890	0.7233
19	672.6588	1399.9393	0.7303	0.1504	0.1132	0.7440
20	662.8772	1401.9342	0.8399	0.1202	0.0703	0.7534

Figure 3 illustrates trends across epochs by showing the evolution of Exact Match, WER, BLEU, and F1-Score metrics. Colored background zones highlight the major training phases: initial learning (green), instability (red), recovery (yellow), and convergence (blue). This long-term consistency in performance metrics and loss values confirms that the model not only converged but also generalized well across increasingly complex normalization tasks. The observed training stability and metric convergence validate the effectiveness of the Dual-BERT architecture in learning context-aware normalization of non-standard Hausa text despite the inherent variability and low-resource challenges of the language.

**Figure 3:**
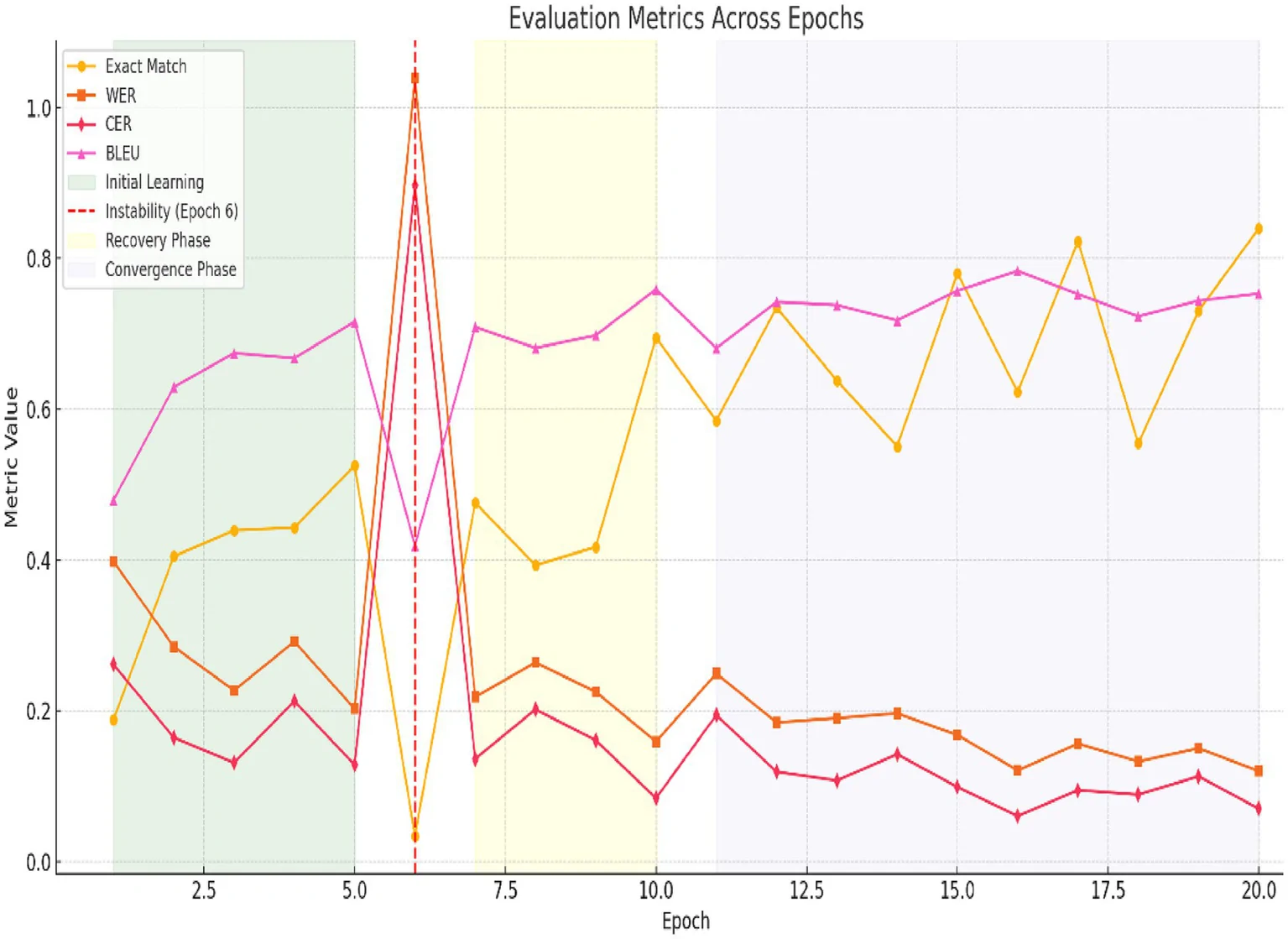
Evaluation metrics across epochs.

### 5.1 Generator vs. discriminator loss across epochs

A critical aspect of the Dual-BERT adversarial training framework lies in the dynamic interplay between the generator and discriminator networks. The training trajectory reveals a consistent decline in generator loss, which dropped from 9058.82 in epoch 1 to 662.88 by epoch 20. This substantial reduction indicates that the generator progressively improved its ability to produce normalized text sequences that closely resemble authentic, human-annotated outputs. The decreasing generator loss reflects its increasing success in learning the complex mapping from non-standard to standard Hausa word forms, including the nuances of phonetic variation, abbreviation, and code-switching patterns.

In contrast, the discriminator loss demonstrated a gradual increase, rising from 529.89 at epoch 1 to 1401.93 at epoch 20. This upward trend suggests that as the generator improved in generating high-quality outputs, the discriminator found it increasingly difficult to distinguish between real (ground truth) and generated (predicted) sequences. In adversarial training, such an inverse loss pattern is often interpreted as a positive indicator of convergence, signaling that the generator is successfully “fooling” the discriminator. The Generator loss reported for Epoch 1 (529.89) represents the cumulative cross-entropy loss accumulated across all training mini-batches during the first epoch. Because the model parameters were initialized from a pretrained multilingual BERT model but had not yet adapted to the Hausa normalization task, prediction errors were relatively high during the initial stage of training, resulting in a large cumulative loss. As training progressed, the loss decreased steadily, indicating successful optimization and convergence of the Generator.

This inverse relationship between the two networks’ losses is a hallmark of effective adversarial learning. Early in training, the discriminator typically dominates due to its advantage in distinguishing poor-quality generated samples. However, as training progresses, the generator learns from these discriminative signals and enhances its output quality, eventually reaching a point where its predictions become nearly indistinguishable from the real data. At this stage, the discriminator’s performance deteriorates not due to failure, but because the task itself becomes inherently more challenging. Figure 4 illustrates the Generator and Discriminator loss trends across 20 training epochs for the Dual-BERT adversarial text normalization model. The graph provides a visual representation of the adversarial learning dynamics between the two networks.

**Figure 4:**
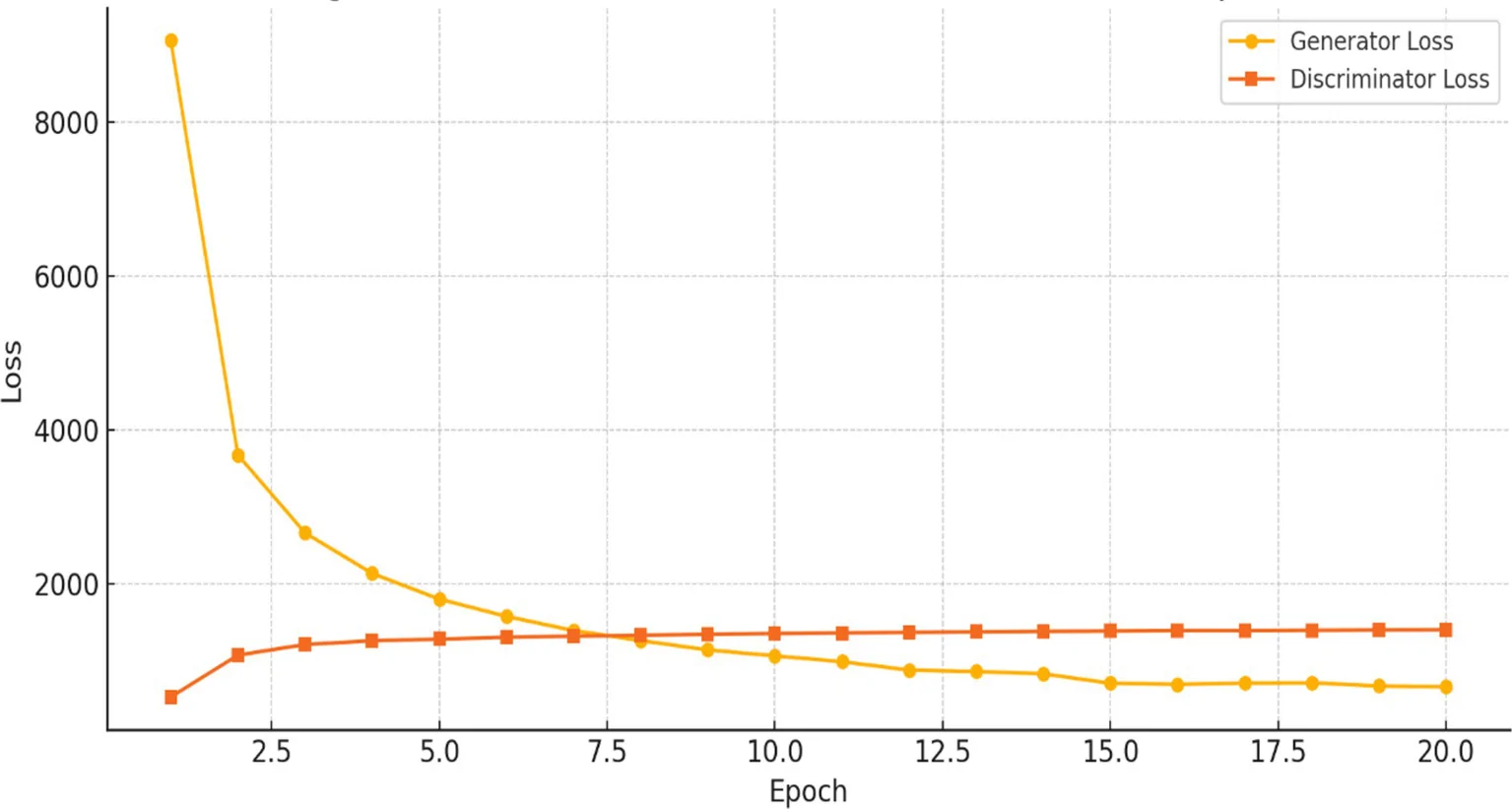
Generator vs. discriminator loss across epochs.

The trends observed in Figure 4 offer greater clarity regarding the dynamics of adversarial training within the Dual-BERT Adversarial framework. The sharp decline in generator losses during the initial epochs indicates that the generator quickly adapted to the feedback from the discriminator, learning to map noisy and non-standard Hausa inputs into more standardized forms. This rapid early improvement reflects the effectiveness of the BERT-based architecture in capturing linguistic dependencies and contextual cues, even in low-resource settings. As training progressed, the generator loss continued to decrease at a slower rate, eventually starbilizing, which suggests that the model approached its optimal representational capacity and reached a stage of convergence.

### 5.2 Candidate performance by accuracy

The accuracy of the candidate generation module was evaluated across different types of textual errors commonly found in noisy or informal input. The system was assessed based on the proportion of test cases where the top 1 candidate matched the standard normalized form, providing a strict measure of its precision. Table 8 presents the number of test words per error category, the number of correct top 1 predictions, and the corresponding accuracy percentage

**Table 8:** Candidate generation performance by accuracy.

Error type	Test words	Correct prediction	Accuracy (%)
Phonetic Substitution	100	89	89%
Deletion	80	65	81.3%
Insertion	70	52	74.3%
Character Elongation	40	32	80%
Abbreviations	30	27	90%
Overall			82.9%

The highest accuracy was observed for abbreviations (90%) and phonetic substitutions (89%), indicating the model’s strong ability to generate correct top 1 suggestions for these types of noisy inputs. Deletion (81.3%) and character elongation (80%) also showed good performance, suggesting robustness in handling character-level variations. The lowest accuracy (74.3%) was noted in insertion errors, where additional characters may introduce more ambiguity, making exact top 1 prediction more challenging. The overall accuracy across all error types was 82.9%, reflecting a high level of correctness in candidate generation when evaluated strictly at rank 1.

### 5.3 Qualitative error analysis

A qualitative analysis provides deeper insights into the strengths and limitations of the proposed approach by examining representative examples of correctly and incorrectly normalized Hausa user-generated content (UGC). Specifically, this analysis focuses on common error categories encountered in informal Hausa text, including Hausa-English code-mixing, dialectal variations, acronyms and abbreviations, character elongations, and insertion/deletion errors. By comparing the model’s predictions with the corresponding reference normalizations, we identify the linguistic phenomena that are effectively handled by the proposed model as well as those that remain challenging. This analysis complements the quantitative results and provides practical evidence of the model’s contextual understanding and areas for future improvement. Table 9 present some examples of qualitative error analysis conducted for the proposed Dual-BERT adversarial network.

**Table 9:** Qualitative error analysis of the Dual-BERT adversarial network.

Error category	Noisy Hausa UGC input	Reference (ground truth)	Model output	Result	Observation
Code-mixed Hausa-English	*zan je meeting gobe in shaa Allah*	*zan je taro gobe in shaa Allah*	*zan je taro gobe in shaa Allah*	✓ Correct	Correctly replaced the English word *meeting* with its Hausa equivalent using contextual information.
Code-mixed Hausa-English	*pls ka turo min file din*	*don Allah ka turo min fayil din*	*don Allah ka turo min file din*	✖ Partial	Successfully expanded *pls* but retained the English word *file*, showing limited handling of embedded English nouns.
Dialectal Variant	*yanzu zamu tafi*	*yanzu za mu tafi*	*yanzu za mu tafi*	✓ Correct	Successfully normalized dialectal spelling and restored standard word segmentation.
Dialectal Variant	*mun gama komai yanzu dai*	*mun gama komai yanzu dai*	*mun gama komai yanzu dai*	✓ Correct	Preserved an already acceptable dialectal expression without unnecessary modification.
Acronym/Abbreviation	*AS sosai*	*Allah ya saka sosai*	*Allah ya saka sosai*	✓ Correct	Correctly expanded a common Hausa abbreviation using contextual cues.
Acronym/Abbreviation	*DSS akwai bayanin nan*	*da sauransu akwai bayanin nan*	*DSS akwai bayanin nan*	✖ Failed	Failed to expand a less frequent abbreviation because of limited contextual evidence in the training data.
Character Elongation	*Nagodeeeee sosai*	*Nagode sosai*	*Nagode sosai*	✓ Correct	Removed repeated characters while preserving the intended lexical meaning.
Character Elongation	*gaskiaaaa ce*	*gaskiya ce*	*gaskiya ce*	✓ Correct	Successfully normalized elongated vowels to standard Hausa orthography.
Insertion Error	*ina sonn zuwa kasuwa*	*ina son zuwa kasuwa*	*ina sonn zuwa kasuwa*	✖ Failed	Failed to remove the inserted character, illustrating the lower performance observed for insertion errors.
Deletion Error	*zamu je skw*	*za mu je kasuwa*	*za mu je sakuwa*	✖ Failed	Recovered the sentence structure but incorrectly reconstructed the deleted lexical item due to insufficient contextual evidence.

### 5.4 Overall model performance

The overall model performance was measured using the following evaluation metrics: Exact-match, BLEU, WER and CER. These metrics provide a holistic assessment of the model’s ability to accurately normalize noisy inputs at both the word and character levels, while also accounting for semantic similarity. The result in Table 9 indicates the overall model performance over 20 epochs.

The overall performance results in Table 10 demonstrate that the Dual-BERT adversarial model achieved strong accuracy and reliability in normalizing noisy Hausa user-generated texts. The exact-match accuracy of 83.99% highlights the model’s ability to produce outputs that are identical to the gold-standard reference in a large majority of cases. Complementing this, the BLEU score of 0.7534 indicates that even in cases where the predictions were not exact matches, the generated sequences closely aligned with reference in terms of semantic meaning, word order, and contextual fluency. Furthermore, the low WER of 12.02% and CER of 7.03% demonstrate that the model introduced only minimal errors at both the word and character levels, ensuring robustness against spelling variations, phonological distortions, and informal orthographic conventions. Taken together, these results confirm that the model not only achieved high token-level precision but also preserved sentence-level meaning and readability, making it highly effective for practical applications such as machine translation, sentiment analysis, speech-to-text, and other downstream NLP understanding tasks in Hausa. Finally, the findings demonstrate that the Dual-BERT Adversarial Network provides a significant advancement in text normalization for low-resource languages, achieving high accuracy and improved generalization on noisy Hausa user-generated content.

**Table 10:** Overall model performance.

Metric	Score
Exact-match	83.99%
BLEU	0.7534
WER	12.02%
CER	7.03%

### 5.5 Baseline comparison

This report compares the performance of three different text normalization models: Transformer S2S, mBERT, mT5, and the proposed Dual-BERT Adversarial Network. The evaluation focuses on four key metrics: Exact Match, WER, CER, and BLEU Score, which collectively assess the models’ accuracy and linguistic fidelity in text normalization tasks. Table 10 presents a comparative evaluation of the proposed Dual-BERT GAN model against baseline models.

Table 11 presents the comparative performance of the proposed Dual-BERT Adversarial Network Model against the baseline Transformer Seq2Seq, mBERT, and mT5 models. The proposed Dual-BERT Adversarial Network Model achieved the highest Exact Match (EM) score of 0.80, outperforming Transformer Seq2Seq (0.68), mBERT (0.70), and mT5 (0.74). Compared with the strongest baseline (mT5), the proposed model improved the Exact Match score by 0.06 (6 percentage points), while achieving a 0.12 (12 percentage points) improvement over the conventional Transformer Seq2Seq model. This demonstrates the effectiveness of combining contextual representations from multilingual BERT with adversarial learning for producing fully correct normalized sentences.

**Table 11:** Comparison evaluation with baseline models.

Model	Exact-match	WER	CER	BLEU
Transformer S2S	0.68	0.31	0.20	0.62
mBERT	0.70	0.22	0.15	0.65
mT5	0.74	0.18	0.12	0.68
Dual-BERT Adversarial Network Model	0.80	0.12	0.07	0.75

A similar trend is observed for Word Error Rate (WER), where lower values indicate better performance. The proposed Dual-BERT Adversarial Network Model achieved the lowest WER of 0.12, compared with 0.31 for Transformer Seq2Seq, 0.22 for mBERT, and 0.18 for mT5. Relative to the Transformer Seq2Seq baseline, this corresponds to approximately a 61.3% reduction in word-level errors, while also representing a 33.3% reduction compared with mT5. These results indicate that the proposed adversarial framework substantially improves word-level normalization accuracy.

The Character Error Rate (CER) results further confirm the superiority of the proposed approach. Dual-BERT Adversarial Network Model achieved the lowest CER of 0.07, compared with 0.20, 0.15, and 0.12 for Transformer Seq2Seq, mBERT, and mT5, respectively. This represents a 65.0% reduction in character-level errors compared with Transformer Seq2Seq and a 41.7% reduction relative to mT5, demonstrating the model’s ability to produce more precise character-level corrections and improved lexical alignment with the reference text.

The proposed model also achieved the highest BLEU score of 0.75, exceeding Transformer Seq2Seq (0.62), mBERT (0.65), and mT5 (0.68). The improvement of 0.07 over the strongest baseline (mT5) indicates better n-gram overlap and stronger syntactic and semantic similarity between the normalized outputs and the reference sentences.

Generally, the Dual-BERT Adversarial Network Model consistently outperformed all baseline models across every evaluation metric. While mBERT and mT5 benefited from contextual representations learned through multilingual pre-training, the integration of dual multilingual BERT encoders with adversarial learning enabled the proposed model to generate more accurate and contextually appropriate normalized text. These results demonstrate that adversarial fine-tuning effectively enhances the contextual modeling capability of multilingual BERT, making the proposed Dual-BERT Adversarial Model the most effective approach for context-aware Hausa user-generated text normalization among the evaluated models

## 6 Conclusion

This study presented a Dual-BERT Adversarial Learning framework for context-aware normalization of Hausa user-generated content. To support this research, a manually annotated parallel corpus comprising noisy and normalized Hausa text collected from Twitter and WhatsApp was developed. The proposed framework combines multilingual BERT contextual representations with adversarial learning to effectively normalize noisy text containing spelling variations, abbreviations, phonetic spellings, acronyms, character elongations, typographical errors, insertion and deletion errors, and Hausa–English code-mixed expressions. Experimental results demonstrate that the proposed model consistently outperformed the evaluated baseline models, achieving an Exact Match Accuracy of 83.99%, BLEU score of 0.7534, Word Error Rate (WER) of 12.02%, and Character Error Rate (CER) of 7.03%. Furthermore, the qualitative analysis showed that the model effectively handles context-dependent normalization challenges and substantially improves the quality of Hausa user-generated text for downstream NLP applications.

Despite these encouraging results, several limitations should be acknowledged. First, although the developed corpus is one of the largest manually annotated Hausa text normalization datasets currently available, it is still relatively small compared with datasets available for high-resource languages. Second, the corpus was constructed primarily from Twitter and WhatsApp data, which may not fully represent linguistic variations occurring on other social media platforms or in different communication domains. Third, although careful annotation guidelines and quality-control procedures were followed, some normalization decisions remain inherently ambiguous because multiple valid standard forms may exist depending on context. Fourth, the proposed model was evaluated only on Hausa user-generated content and its generalization to unseen platforms, emerging slang, or other low-resource languages has not yet been investigated. Finally, while ethical measures including informed consent for WhatsApp data collection and anonymization of personally identifiable information were implemented, the use of user-generated content continues to require careful attention to privacy, consent, and responsible data governance.

Future work will focus on expanding the corpus to include additional social media platforms and broader dialectal coverage, investigating larger multilingual and language-specific pretrained language models, conducting comprehensive ablation studies to further quantify the contribution of each architectural component, and evaluating the proposed framework across additional downstream NLP tasks such as machine translation, sentiment analysis, named entity recognition, and question answering. These directions will further improve the robustness, scalability, and applicability of context-aware text normalization for Hausa and other low-resource languages.

## Data Availability

Publicly available datasets were analyzed in this study. This data can be found at: https://github.com/hausanlp/NaijaSenti/blob/main/data/annotated_tweets/hau/dev.tsv.
